# Supplementary material for: Unveiled electric profiles within hydrogen bonds suggest DNA base pairs with similar bond strengths
Source: PLoS One. 2017 Oct 5;12(10):e0185638. doi: 10.1371/journal.pone.0185638 (PMC5628848; doi:10.1371/journal.pone.0185638)
Supplement: S1 File — (PDF) [file pone.0185638.s001.pdf]

S1. On the design of our DFT method.

In the frame of the computational approach used for the electrostatic calculations inside bonds, we develop a DFT method, based on a methodology previously introduced by some of us[1-3]. This methodology justifies the use of static and mean field DFT calculations to approach non-equilibrium quantities as **E**-field response. The method provides self-consistent exchange-correlation potentials to solve intrinsic many-body problems while retaining the advantages of the mean-field approach. Technically, we develop a self-consistent procedure to develop adjustable V_{xc} for "each" numerical *ab-initio* calculation required.

Our computational method is supported by previous accepted works regarding i.) the existence of nonlocal V_{xc} to manage DFT calculations within static approaches[4,5] and ii.) the design of nonlocal V_{xc} within mean-field approach to attain many-body effects[1,5] and (d) the definition of a nonlocal functional to sustain the approaches as such here developed[3]. A brief summary of the general scheme for the exchange-correlation potential definition by us developed is below summarized.

By using arguments/theorems of DFT we could safely guarantee that any quantity can be modeled and computed remaining within mean field density representations,

$$\boxed{\left\langle \Psi(\vec{r},\vec{r}_2,\ldots,\vec{r}_N)\right|\hat{O}\left|\Psi(\vec{r},\vec{r}_2,\ldots,\vec{r}_N)\right\rangle = O[n(\vec{r})]}$$

as we will sketch here. We define in each case our static mean-field problem,

$$\boxed{H_{mean-field}\ \varphi_i(\vec{r}_i) = \varepsilon_i\ \varphi_i(\vec{r}_i)} \qquad \text{(S3)}$$

where the Hamiltonian within DFT formalism is conveniently separated as,

$$\boxed{\left[-\frac{1}{2}\nabla^2 + V_{ext} + V_{xc}(\vec{r}_i)\right]\varphi_i(\vec{r}_i) = \varepsilon_i\ \varphi_i(\vec{r}_i)}$$

The approach required to evaluate the quantities we are interested in (e.g. **E**-field and total energies) relies on the definition of a nonlocal V_{xc} that introduce adjustable nonlocality as a mean to approach exactness within static DFT [details in ref. 5 and our ref. 1],

$$\boxed{V_{xc}^{HF-DFT} = a\,V_x^{HF} + (1-a)V_x^{DFT} + V_c^{DFT}} \tag{S4}$$

The boundary conditions used to complete the definition of V_{xc} involve the application of some mathematical properties connecting Green Functions, eq. 1, and eigenstates, eq. 2, i.e. The poles of the non-interacting Green Functions must coincide with the highest occupied molecular orbital (HOMO) and with the lowest unoccupied molecular orbital (LUMO) gaps in the single particle DFT scheme. Such analysis leads us to device here a self-consistent procedure to define V_{xc} (details in ref. 1).

To be consistent with the formalism here stated the numerical implementation of DFT functionals, V_x or V_c must correspond with any local DFT functional, see ref. 8 for more insights. In this manuscript we have used PW91 as a seed (i.e. zero nonlocal approach limit) to run our hybrid functionals.

REFERENCES

1. García, Y. & Sancho-García, J.C. On the role of the nonlocal Hartree-Fock exchange in *ab-initio* quantum transport: H_2 in Pt nanocontacts revisited. *Journal of Chemical Physics* **129**, 034702 (2008).

2. García, Y. Influence of CO in the structural and electrical properties of Pt nanocontacts: a comparison with H2 molecules addition. *Journal of Chemical Physics* **131**, 014702 (2009).

3. García, Y., Cuffe, J., Alzina, F. and Sotomayor-Torres, C. M. Non local correction to the electronic structure of non ideal electron gases: the case of graphene and tyrosine amino acid. *Journal of Modern Physics* **4-4**, 522 - 527 (2013).

4. Hohenberg, P. & Kohn, W. Inhomogeneous Electron Gas. *Phys. Rev. B*. **136**, 864-871 (1964).

5. R. K. Nesbet. Beyond Density Functional Theory: The domestication of nonlocal potentials. *Modern Physics Letters B* **18**, 73 (2004).

6. Mishima O. & Stanley, H. E. The relationship between liquid, supercooled and glassy water. *Nature* **396**, 329-335 (1998).

7. Muller-Dethlefs, K. & Hobza, P. Noncovalent interactions: A challenge for experiment and theory. *Chemical Reviews* **100**, 143-167 (2000).

8. Ludwig, R. Water: From clusters to the bulk. *Angewandte Chemie-International Edition* **40**. 1808-1827 (2001).

9. Kohn, W. and Sham, L. J.. Self-Consistent Equations Including Exchange and Correlation Effects. *Physical Review* **140**, 1133–1138 (1965).

10. Perdew, J. P. and Wang, Y. Accurate and simple density functional for the electronic exchange energy: Generalized gradient approximation. *Physical Review B* **33**, 8800-8802 (1986).

11. Perdew, J.P. *.et. al*. Atoms, molecules, solids, and surfaces: Applications of the generalized gradient approximation for exchange and correlation. *Physical Review B* **46**, 6671-6687 (1992).

12. Staroverov, V. N. ,Scuseria, G. E. ,Tao, J. and Perdew, J. P.. Comparative assessment of a new nonempirical density functional: Molecules and hydrogen-bonded complexes. *Journal of Chemical Physics* **119**, 12129-12137 (2003).

13. Sobczyk, L., Grabowski, SJ. & Krygowski, TM. Interrelation between H-bond and Pi-electron delocalization . *Chemical Reviews* **105**, 3513-3560 (2005).

14. Arunan, E. *et. al.* Defining the hydrogen bond: An account (IUPAC Technical Report). *Pure and Applied Chemistry* **83**, 1619-1636 (2011).

15. Steiner, T. The Hydrogen Bond in the Solid State. *Angewandte Chemie-International Edition* **41**, 48-76 (2002).

16. Todeschini, R. and V. Consonni. Handbook of Molecular Descriptors, Wiley-VCH Verlag GmbH (2008).
