# Supplementary material for: Unveiled electric profiles within hydrogen bonds suggest DNA base pairs with similar bond strengths
Source: PLoS One. 2017 Oct 5;12(10):e0185638. doi: 10.1371/journal.pone.0185638 (PMC5628848; doi:10.1371/journal.pone.0185638)
Supplement: S2 File — (PDF) [file pone.0185638.s002.pdf]

S2. Understanding electrical forces in the ubiquitous water dimer.

The water dimer offers a feasible and comprehensive frame to study hydrogen bonds (HB)[6-8]. Actually, 2●(H_2O) is a manageable system to understand the impact of numerical approximations on final numerical outcomes and to rationalize the response of HB relative to coexistent polar covalent bonds (CB).

a

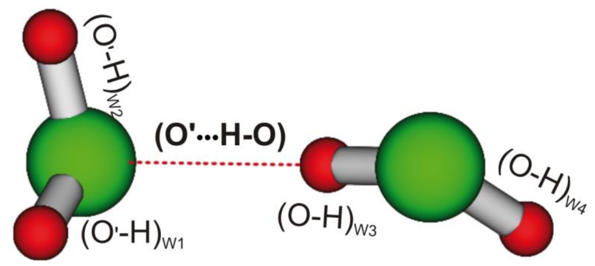

b

Bond Label	Bond Distance (Å)
(O'•••H-O)	1.90
$(O'\text{-}H)_{W1}$	0.96
$(O'\text{-}H)_{W2}$	0.96
$(O\text{-}H)_{W3}$	0.97
$(O\text{-}H)_{W4}$	0.96

Figure S1. Schematic representation of the water dimer. a, By green and red colours we represent oxygen and hydrogen atoms respectively. HB pattern is represented by red dashed lines. **b**, Geometric distances for all the bonds are included in the table.

Fig. S1 includes the scheme of the geometry we use to perform numerical calculations. The structure is the configuration we obtain after fully relaxing the system with 6-31g (d,p) basis set and using an hybrid exchange-correlation potential (V_{xc}) to introduce many-body electron-electron interactions within a reduced mean-field model[9-12]. The V_{xc} is modelled following the methodology already developed by some of us[1]. Here, V_{xc} is defined as 80% PW91[10] and 20% Fock for exchange contributions (a summary on the method to design V_{xc} is provided as part of Supp. Mat. 3).

Fig. S2 is indicative of the behaviour of the **E**- field along the axis defined by the HB (O···H) in the water dimer. In a similar trend, Fig. S3 reports **E**-profile of the covalent bond $(OH)_{W1}$ (see Fig.1 for spatial representation of the bonds). The CB depicts a larger gradient in the variation of **E**.

The angle between **E** vector and HB axis is named Φ. The behaviour of the function $\Phi \equiv \Phi(d)$ is represented in the lower panels in Figure´s S2 and S3. It resembles the profile of two positive

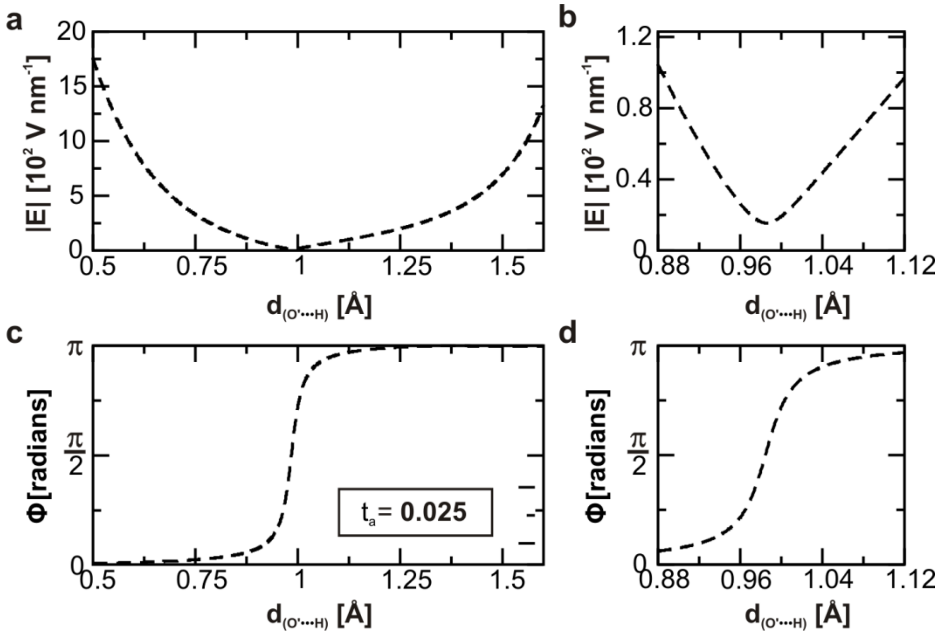

Figure S2. Electrical characterization of HBs for the water dimer. a, Representation of the variation of the electric field modulus (|**E**|) in the two atoms axis. The asymptotic behaviour coincides with O and H positions (positive charge centres in our model). **b**, Characterization of the evolution of the angle between **E** and the two atom axis, Φ= Φ(d). The deviations of such curves from the ideal Heaviside function are an indication of the electric susceptibility of chemical bonds. The descriptor value included in the inset accounts for this information.

poles instead of a dipole model. This argument supports the claim in favour of a modified electrostatic picture for chemical bonds[13]. The use of the index introduced in Supp. Mat. S3 for the description of $\Phi \equiv \Phi(d)$ facilitates the understanding of such electrostatic features and establishes a frame to quantify the electrical feasibility of atomic bonds.

Table S1 lists the values of the indexes for all the oxygen-hydrogen bonds in the water dimer. The values are normalized to the index obtained for the archetypal HB of the water dimer. The

two-orders of magnitude that differentiate CB indexes from HB index are indicative of the higher electrical inertia of the CB when comparing with HB.

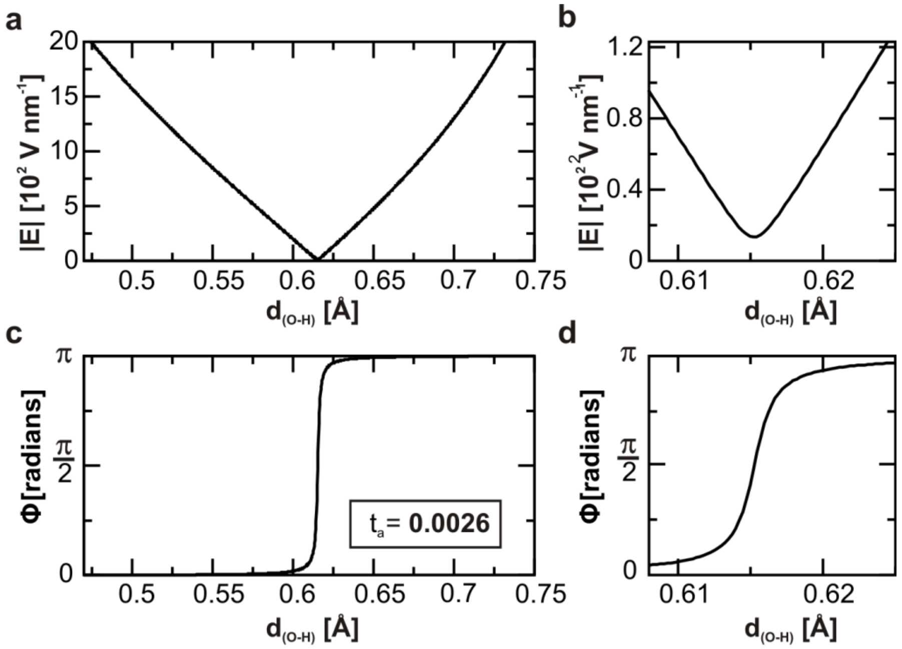

Figure S3. Electrical characterization of CB for the water dimer. a and **b,** Representation of the variation of the electric field modulus (|**E**|) along the bond axis. The asymptotic behaviours coincide with O and H positions (positive charge centres in our model). **c** and **d**, Characterization of the evolution of the angle between **E** and the interaction axis, along the bond length, Φ= Φ(d). The deviations of such curves from the ideal Heaviside function are an indication of the electric susceptibility of chemical bonds. The descriptor included in the insets accounts for this information.

Fig. S4 is a summary of the analysis carried out to estimate errors in numerical results. Regarding the *ab-initio* models we highlight the imperceptible influence of the exchange model

**Bond Label**	$t_a / t_a(O' \cdots H)$
**O-H$_{W1}$**	0.025
**O-H$_{W2}$**	0.024

**O-H$_{W3}$**	0.024
**O-H$_{W4}$**	0.025

Table S1. Numerical results for the electric field descriptor, t_a, describing O-H covalent bonds in the water dimer. Results are normalized to the HB descriptor in the dimer.

in the mean field potentials and the completeness of the basis set as shown in Figure´s S4a and S4c. This result agrees with the fulfilment of the Hellmann-Feynman theorem for electrostatic forces and its relation with the selection of the exchange-correlation potential within *ab-initio* models. In regards with basis sets selection we have applied an accepted reasoning in our field, we increase the basis sets size until we fix the error below a selected limit[1].

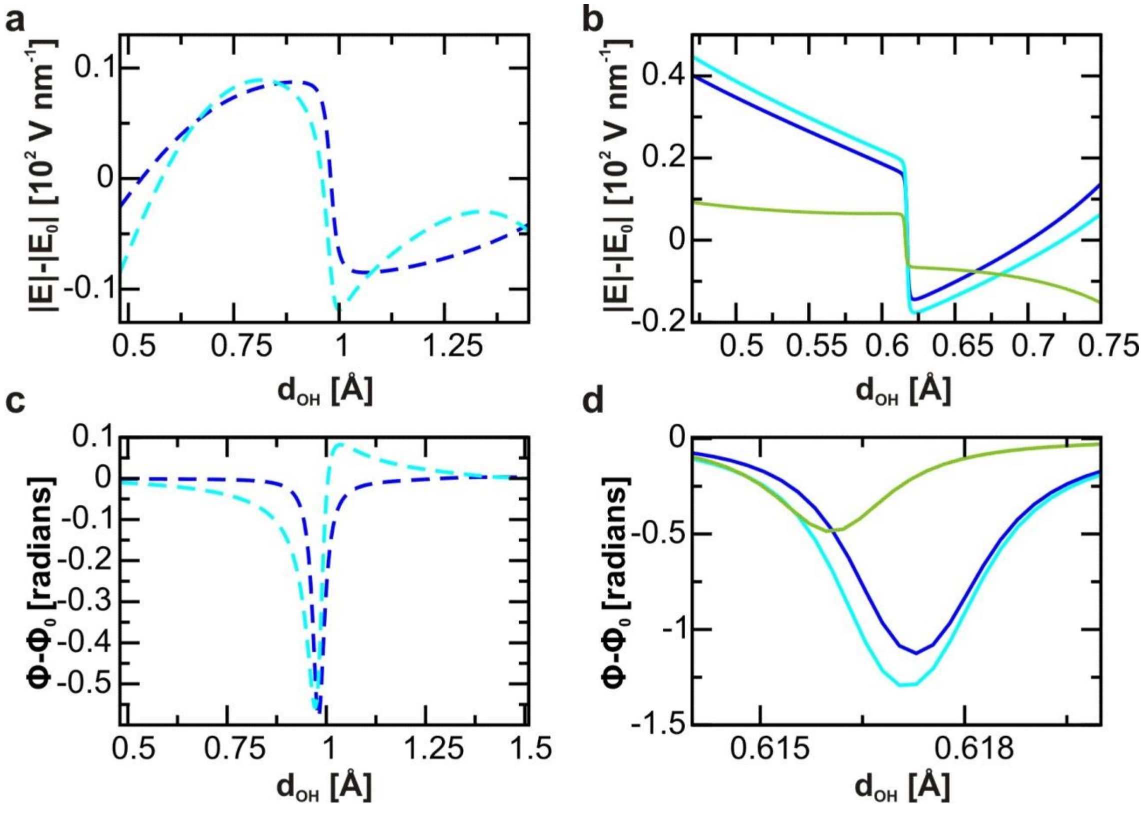

Figure S4. Estimation of the errors introduced by *ab-initio* methods. We report the deviation of the |**E**| and the Φ values relative to values reported in Figs. 2 and 3. The influence of mean-field potentials and as well as basis sets completeness for HB is represented in the panels **4a** and **4c**, and for the selected CB (O´-H)$_{W1}$ is indicated in **4b**

and **4d**. In dark blue colour we represent the mean value deviations when the Fock exchange is 80 % of the total exchange. It represents an upper bar to the effect of the exchange in the model outcomes. As noticed in all the cases, the errors remain below 1%. By light blue colour we represent mean value deviations when 6-311g basis set is used. Relative errors remain below 10%. This higher value corresponds with the singular points, the poles in the eq. S2. Then, such points will not affect the numerical results which accounts for a limit in this equation.

REFERENCES

1. García, Y. & Sancho-García, J.C. On the role of the nonlocal Hartree-Fock exchange in *ab-initio* quantum transport: H_2 in Pt nanocontacts revisited. *Journal of Chemical Physics* **129**, 034702 (2008).

2. García, Y. Influence of CO in the structural and electrical properties of Pt nanocontacts: a comparison with H2 molecules addition. *Journal of Chemical Physics* **131**, 014702 (2009).

3. García, Y., Cuffe, J., Alzina, F. and Sotomayor-Torres, C. M. Non local correction to the electronic structure of non ideal electron gases: the case of graphene and tyrosine amino acid. *Journal of Modern Physics* **4-4**, 522 - 527 (2013).

4. Hohenberg, P. & Kohn, W. Inhomogeneous Electron Gas. *Phys. Rev. B*. **136**, 864-871 (1964).

5. R. K. Nesbet. Beyond Density Functional Theory: The domestication of nonlocal potentials. *Modern Physics Letters B* **18**, 73 (2004).

6. Mishima O. & Stanley, H. E. The relationship between liquid, supercooled and glassy water. *Nature* **396**, 329-335 (1998).

7. Muller-Dethlefs, K. & Hobza, P. Noncovalent interactions: A challenge for experiment and theory. *Chemical Reviews* **100**, 143-167 (2000).

8. Ludwig, R. Water: From clusters to the bulk. *Angewandte Chemie-International Edition* **40**. 1808-1827 (2001).

9. Kohn, W. and Sham, L. J.. Self-Consistent Equations Including Exchange and Correlation Effects. *Physical Review* **140**, 1133–1138 (1965).

10. Perdew, J. P. and Wang, Y. Accurate and simple density functional for the electronic exchange energy: Generalized gradient approximation. *Physical Review B* **33**, 8800-8802 (1986).

11. Perdew, J.P. *.et. al*. Atoms, molecules, solids, and surfaces: Applications of the generalized gradient approximation for exchange and correlation. *Physical Review B* **46**, 6671-6687 (1992).

12. Staroverov, V. N. ,Scuseria, G. E. ,Tao, J. and Perdew, J. P.. Comparative assessment of a new nonempirical density functional: Molecules and hydrogen-bonded complexes. *Journal of Chemical Physics* **119**, 12129-12137 (2003).

13. Sobczyk, L., Grabowski, SJ. & Krygowski, TM. Interrelation between H-bond and Pi-electron delocalization . *Chemical Reviews* **105**, 3513-3560 (2005).

14. Arunan, E. *et. al.* Defining the hydrogen bond: An account (IUPAC Technical Report). *Pure and Applied Chemistry* **83**, 1619-1636 (2011).

15. Steiner, T. The Hydrogen Bond in the Solid State. *Angewandte Chemie-International Edition* **41**, 48-76 (2002).

16. Todeschini, R. and V. Consonni. Handbook of Molecular Descriptors, Wiley-VCH Verlag GmbH (2008).
