# Supplementary material for: Unveiled electric profiles within hydrogen bonds suggest DNA base pairs with similar bond strengths
Source: PLoS One. 2017 Oct 5;12(10):e0185638. doi: 10.1371/journal.pone.0185638 (PMC5628848; doi:10.1371/journal.pone.0185638)
Supplement: S3 File — (PDF) [file pone.0185638.s003.pdf]

S3. Definition of a numerical descriptor for a quantification of the electrical forces in directional bonds.

Our manuscript is focused on the electrical and mechanical description of HB in DNA with intrabond resolution. Such features, according to the basic Density-Functional Theory (DFT) principles[4,9], could be obtained by developing an exact approach based upon the exclusive knowledge of the electron density distribution in the HB regions (according to DFT principles all effects derived from -apparently- external events are included in the electron density distribution). Consequently the characterization of the susceptibility of these HB exclusively depends on the internal response of the HB´s electron cloud. However, there exists a void of knowledge related to classical approaches, in terms of structural/electrical parameters, that can be useful to characterize the behavior of the interactions within the bond region[14,15]. Probably, this absence of theoretical tools is a consequence of that even today most of the phenomena of interest for Science can be described using existing classical approaches, e.g. the dipole model,

or quantum mechanics approaches e.g. to estimate the energy and the structure of atomic systems.

We here define a numerical descriptor[16] (labelled as 't_a') in order to study the effect of external fields on the electromechanical response of bonded atoms. This descriptor accounts for the deviations of the **E**-field along the bond length relative to the behaviour expected for two positive point charges immersed in an uniform electron density region (see Fig. 1 in the main manuscript).

We investigate the dependence between the intrabond position (d) and the angle between **E** and the intrabond axis (Φ) , i.e. $\Phi = \Phi(d)$. For ideal behaviours, such dependence is represented by a Heaviside transition between 0º and 180º, in the region between the two extreme atom positions,

$$\Phi_{ideal}(d) = \lim_{t \to 0} \left[\frac{\pi}{2} + \tan^{-1}\left(\frac{(d - d_0)/a_0}{t/a_0} \right) \right] \quad \text{(S1)}$$

In the expression above a_0 is the Bohr radius and d_0 represents the point where the 0-180º step transition takes place. In our context, such d_0-point correspond to the location where **E** changes the orientation relative to the two-atom bond axis. We define the dimensionless descriptor as $t_a = \left| \frac{t}{a_0} \right|$ for finite t - valued functions. This reasoning gives us the following expression for t_a ,

$$t_a = \left| \frac{(d - d_0)/a_0}{\tan\left[\Phi(d) - \frac{\pi}{2} \right]} \right| \quad \text{(S2)}$$

We reported t_a values after statistical treatment for a set of N points $(d\ , \Phi(d))$ in each of the studied intrabond interactions. The statistical treatment comprises the fitting of such points to modulated Heaviside functions, eq. S2. The final t_a values, were obtained as the average over nearly a thousand points and the standard deviations for each bond was lower than 10^{-5} .

REFERENCES

1. García, Y. & Sancho-García, J.C. On the role of the nonlocal Hartree-Fock exchange in *ab-initio* quantum transport: H_2 in Pt nanocontacts revisited. *Journal of Chemical Physics* **129**, 034702 (2008).

2. García, Y. Influence of CO in the structural and electrical properties of Pt nanocontacts: a comparison with H2 molecules addition. *Journal of Chemical Physics* **131**, 014702 (2009).

3. García, Y., Cuffe, J., Alzina, F. and Sotomayor-Torres, C. M. Non local correction to the electronic structure of non ideal electron gases: the case of graphene and tyrosine amino acid. *Journal of Modern Physics* **4-4**, 522 - 527 (2013).

4. Hohenberg, P. & Kohn, W. Inhomogeneous Electron Gas. *Phys. Rev. B*. **136**, 864-871 (1964).

5. R. K. Nesbet. Beyond Density Functional Theory: The domestication of nonlocal potentials. *Modern Physics Letters B* **18**, 73 (2004).

6. Mishima O. & Stanley, H. E. The relationship between liquid, supercooled and glassy water. *Nature* **396**, 329-335 (1998).

7. Muller-Dethlefs, K. & Hobza, P. Noncovalent interactions: A challenge for experiment and theory. *Chemical Reviews* **100**, 143-167 (2000).

8. Ludwig, R. Water: From clusters to the bulk. *Angewandte Chemie-International Edition* **40**. 1808-1827 (2001).

9. Kohn, W. and Sham, L. J.. Self-Consistent Equations Including Exchange and Correlation Effects. *Physical Review* **140**, 1133–1138 (1965).

10. Perdew, J. P. and Wang, Y. Accurate and simple density functional for the electronic exchange energy: Generalized gradient approximation. *Physical Review B* **33**, 8800-8802 (1986).

11. Perdew, J.P. *.et. al*. Atoms, molecules, solids, and surfaces: Applications of the generalized gradient approximation for exchange and correlation. *Physical Review B* **46**, 6671-6687 (1992).

12. Staroverov, V. N. ,Scuseria, G. E. ,Tao, J. and Perdew, J. P.. Comparative assessment of a new nonempirical density functional: Molecules and hydrogen-bonded complexes. *Journal of Chemical Physics* **119**, 12129-12137 (2003).

13. Sobczyk, L., Grabowski, SJ. & Krygowski, TM. Interrelation between H-bond and Pi-electron delocalization . *Chemical Reviews* **105**, 3513-3560 (2005).

14. Arunan, E. *et. al.* Defining the hydrogen bond: An account (IUPAC Technical Report). *Pure and Applied Chemistry* **83**, 1619-1636 (2011).

15. Steiner, T. The Hydrogen Bond in the Solid State. *Angewandte Chemie-International Edition* **41**, 48-76 (2002).

16. Todeschini, R. and V. Consonni. Handbook of Molecular Descriptors, Wiley-VCH Verlag GmbH (2008).
